# Onychomadesis in a 9-month-old boy with hand-foot-mouth disease

**DOI:** 10.1186/s12245-017-0152-9

**Published:** 2017-08-14

**Authors:** Ibrahim Mortada, Rola Mortada, Mohamad Al Bazzal

**Affiliations:** Independent Consultant, Beirut, Lebanon

**Keywords:** HFMD, Onychomadesis, Nail shedding, Infectious diseases, Dermatology

## Abstract

**Background:**

Nail abnormalities in childhood are generally uncommon. Recently, onychomadesis is described as a rare, late complication of hand-foot-mouth disease, which is a viral illness commonly seen in the pediatric age group. It is therefore important to elucidate the presentation of this entity, especially in the context of the hand-foot-mouth disease.

**Case presentation:**

We report a case of onychomadesis in a 9-month old Lebanese boy who presented to the emergency department with rapidly progressing nail changes involving all four extremities. These changes appeared few days after the healing of cutaneous lesions of hand-foot-mouth disease.

**Conclusions:**

This case highlights the importance of recognizing the association between onychomadesis and hand-foot-mouth disease in order to avoid unnecessary treatment and to reassure the patient’s parents.

## Background

Onychomadesis, characterized by shedding of nails from the proximal nail beds, is often idiopathic, but can also be linked to certain medications, systemic illnesses, and viral infections including hand-foot-mouth disease (HFMD). HFMD is a common contagious disease, affecting mainly children under the age of 10, but also reported in adults [1]. This disease is characterized by vesicular rashes on hands, feet, and buttocks and ulcers in the oral mucosa, accompanied by occasional fever [2]. Enterovirus 71 and coxsackievirus A16 are the most common causative agents associated with the condition [3]. Onychomadesis, a rare complication occurring 4 to 6 weeks after the onset of the disease, is usually self-limited and does not require treatment [4]. Common nail abnormalities include leukonychia and Beau lines as well as partial or complete nail shedding [5]. The mechanism of onychomadesis remains to be elucidated, although onychomadesis usually implies that nail matrix proliferation was temporarily inhibited. Bettoli et al. suggest that periungual inflammation secondary to viral infection may be induced directly by viruses or indirectly by immunocomplexes and consequent distal embolism [6], while Cabrerizo et al. consider that the nail matrix is directly damaged by viral replication, based on the presence of Coxsackie virus 6 in shed nails [7].

In this paper, we describe the presentation of onychomadesis in a boy previously diagnosed with HFMD.

## Case presentation

**Fig. 1:**
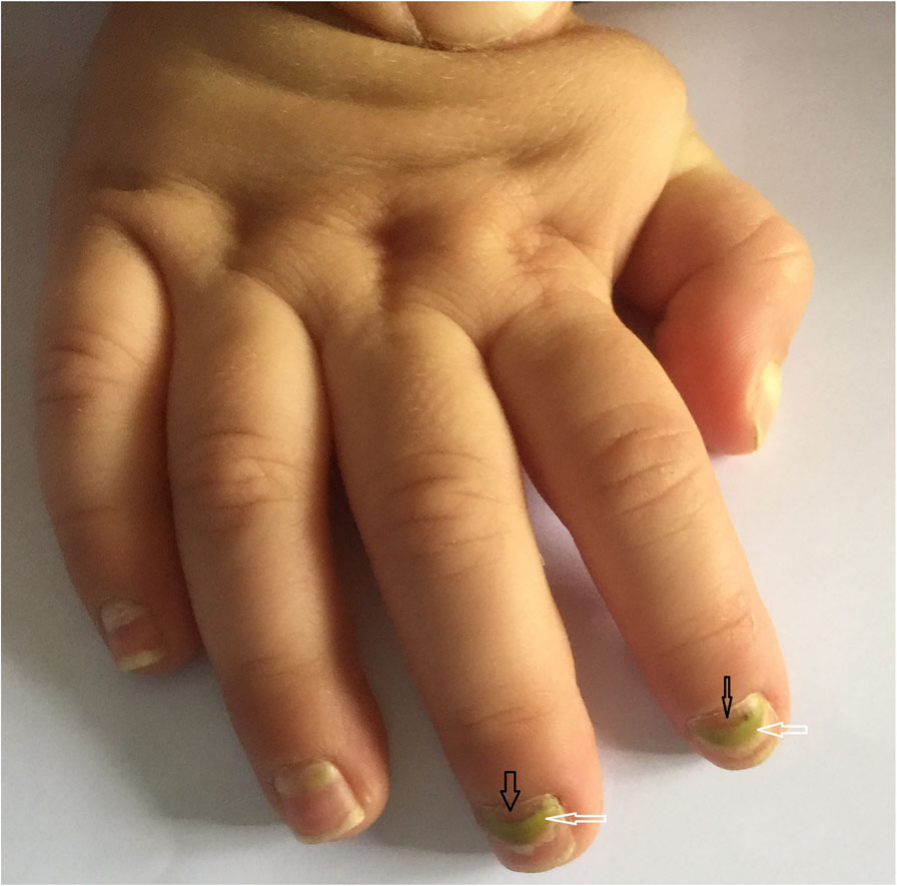
New nail growth (*black arrows*) and *greenish-yellowish* patch (*white arrows*) in *right* hand

**Fig. 2:**
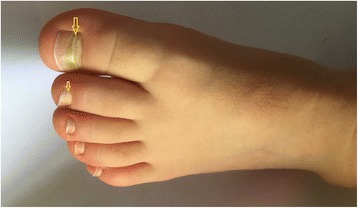
Beau *lines* (*yellow arrows*) in *left* foot

A 9-month-old boy presented to the emergency department at a tertiary health care center in Beirut in September with a two-day history of nail shedding involving all four extremities. The patient began to have changes in the nails 6 days following the resolution of painful vesicles on the hands, feet, and mouth accompanied by mild fever (temperature 38.3 °C), diagnosed as HFMD. The oral blisters had been interfering with feeding; however, the patient did not receive any treatment besides supportive care, leading to the spontaneous resolution of the vesicles. Nail changes began as a greenish-yellowish patch appearing at the beginning of the nails and spreading towards the free edge. The nails then started to shed from the lunula towards the free edge concomitantly with the appearance of slowly growing new, pink nails (Fig. 1). Beau lines, horizontal grooves running across the nail plates, were noted (Fig. 2).

## Conclusions

To our knowledge, this is the first case of onychomadesis to be reported from the Middle East. We ought to highlight the importance of recognizing the association between HFMD and onychomadesis in order to avoid unnecessary treatment and to reassure the patient’s parents. Nevertheless, it is important to note that this is a rare presentation, and a thorough history and physical exam are necessary to identify the correct etiology and rule out other serious pathologies.
